# Measuring wool cortisol and progesterone levels in breeding maiden Australian merino sheep (*Ovis aries*)

**DOI:** 10.1371/journal.pone.0214734

**Published:** 2019-04-08

**Authors:** Gregory Sawyer, Danielle Webster, Edward Narayan

**Affiliations:** School of Science and Health, Western Sydney University, Penrith, Australia; Centre for Cellular and Molecular Biology, INDIA

## Abstract

Hormonal assessment tools are important for determining the reproductive success of production animals. This study used non-invasive wool assessment to quantify changes in progesterone and cortisol levels in reproducing female merino sheep. Wool samples were collected from a group of n = 46 maiden merino ewes (22–25 months old), naturally joined under natural light conditions in southern New South Wales (NSW), Australia. Three shearing opportunities were conducted as part of standard on-farm management practices. The wool samples were collected at three different dates during 2017, January (prior to rams being put out with the mob and to provide a baseline level since previous shearing in May 2016), September (during very late stages of gestation–approximately 2 weeks prior to parturition) and December (ewes had given birth and ~2-month-old lambs were at foot). Analysis of cortisol and progesterone was conducted concurrently from the same sample of wool. The hormones in wool samples quantified using commercially available cortisol and progesterone enzyme-immunoassay kits. Wool cortisol concentrations increased significantly (p = 3.04E-14) from pre-joining in January (1.33±0.12 ng/g) to late gestation in September (3.59±0.12 ng/g). Concentration of wool cortisol post-lambing in December (3.27±0.14 ng/g) did not decline significantly (p = 0.124) after gestation however remained significantly higher (p = 3.82E-10) than pre-joining levels. Wool progesterone (PG) concentrations increased significantly (p = 1.83E-33) from pre-joining (0.04±0.005 ng/g) in January to late gestation in September (5.53±0.13 ng/g) with a significant (p = 5.44E-59) decline observed in December (0.05±0.003 ng/g) to post- pregnancy concentrations. No significant difference was shown between pre-joining and post lambing PG concentrations (p = 0.057). Our results showed that non-invasive assessment of hormones in Merino sheep wool reflected significant increase in both cortisol and progesterone guided by pregnancy.

## Introduction

Measurement of glucocorticoids or stress hormone (cortisol) in blood, faeces and saliva has often been used to measure the physiological responses of sheep to physical and psychological stressors [1]. Analysis of long-term changes in cortisol levels in sheep has historically been difficult to obtain. Evaluation of steroids in non-invasive samples such as sheep wool can provide hormonal indices of reproduction [2]. This field of research is new and it is believed that blood borne steroids are slowly incorporated into the emerging hair/wool shaft and slowly grow out with it, reflecting the steroid level over the growth period for that hair/wool staple [2, 3]. The current study is based on the non-invasive assessment of reproductive and stress hormones in wool samples to provide a rapid biomarker to infer reproductive activity of sheep and assess stress levels during pregnancy in Australian merino ewes.

The hypothalamus and the pituitary have a direct role in the production of both stress and reproductive hormones, making the relationship between the two foreseeable [4]. Reproduction in merino ewes and rams is controlled by the release of gonadotrophin-releasing hormone (GnRH) that is produced in the hypothalamus. GnRH then acts on the anterior pituitary to produce luteinising hormone (LH), which triggers the production of oestrogen (from the ovaries) or testosterone (from the testes) [4].

In female sheep, progesterone (PG) is a hormone that is synthesized by the corpus luteum (a yellow body in the ovary of mammals, where follicular cells develop into corpus luteum) to proliferate the endometrium in preparation for an embryo [5]. It is essential for the establishment and maintenance of pregnancy in all mammals. PG is used during early embryonic development for implantation and placentation and if levels of PG are insufficient, it can lead to failure of the pregnancy and thus early embryonic loss [6] with unsuccessful embryos mostly lost before the critical 13-day stage when maternal recognition occurs and thus does not impact on the length of the ewe’s cycle. After 35 days of gestation, the fetal period begins and continues for approximately 145–150 days until full gestation is completed [7]. PG hormone treatments for sheep can be used to induce cycling in anoestrus ewes. These treatments have been shown to improve pregnancy and conception rates as well as allowing producers to synchronise oestrus in sheep for easier reproductive management [8]. The reproductive system of the ewe is particularly susceptible to stress. This is due to the effects stress has on decreasing the production GnRH and LH, which can either block or delay the development of follicles in the ewe resulting in sub-fertility [9].

Previous research conducted by [10] on nine 3-year-old Corriedale female sheep investigated the effects of water restriction on both blood and wool cortisol levels. The authors concluded that wool cortisol was a better marker of stress than blood cortisol and was suitable for evaluating heat stress in sheep. The advantages of using wool samples to assess stress in sheep include reducing the variations caused by acute stressors and circadian rhythms that can cause inconsistencies in results [10]. Recently, [11] also applied wool cortisol tests in Corriedale ewes to evaluate changes in stress indices in relation to water addition to total mixed ration, finding no significant effects of treatment on stress conditions.

Pregnancy and lactation are known to affect the circulating levels of ovarian steroids including PG and oestradiol in many mammals. Previous research has found that oestradiol rises gradually throughout pregnancy, peaking before parturition and then declining rapidly [12]. Similar to cortisol, the analysis of ovarian steroids often requires invasive sampling techniques to collect blood or saliva. These samples also have their limitations, only capturing a snapshot of a short period of time for the hormones measured. There are a very limited number of studies that use hair to measure long term ovarian steroid levels [12], and none carried out on wool that could be found. Recognising this gap in literature, this research collected wool samples to examine the long-term hormonal function, before, during and after pregnancy in Australian merino sheep.

The aims of this research were two-fold; firstly (1) we tested wool cortisol and progesterone levels in maiden merino ewes taken pre-, during and post-joining. Secondly, (2) we compared levels of both steroids with the time period of pregnancy to determine how wool derived hormones varied with respect to reproduction in merino ewes.

## Material and methods

Western Sydney University (WSU) Animal Care and Ethics Committee approved this study (Protocol number A12345). All research samples were obtained during standard husbandry procedures of the commercial sheep producer.

### Animals and animal handling

The sheep used in this study were all located at the property of Ooranook Pastoral Company, Braidwood, NSW 2622 (GPS-35.251631, 149.867294).

The focus mob consisted of 46 maiden Merino ewes from the same mob born in 2015. The Merino ewes and their offspring in this study were all produced by natural joining. All sheep remained at the Ooranook farm throughout the study from January 2017- December 2017 where all sample collection took place. The rams were introduced to the focus mob on the 26^th^ of April, 2017.

The owner of the research flock undertakes shearing of young sheep (less than two years old) several occasions throughout the first two years of their life as part of their management. This provides ample opportunities for samples to be collected at various points throughout the year from the same sheep. The removal of the wool fibre provides a non-invasive method for the assessment in the laboratory for the correlation of hormone levels, with long term stressors, that may provide insight into causes of reproductive wastage.

The sheep used in this trial were “run with” a larger mob of 100 ewes of the same age, same shearing procedure and of the same blood lines. The sheep were run over one large paddock during the lifetime of the project paddock. The paddock size is 462 acres (187 hectares), with 5 watering points. The feed on offer was native natural grasses local to the district and research paddocks with the naturally occurring grass being weeping grass (*Microlaena stipoides*). The paddock has 34% wooded timber for shelter from the environmental conditions.

The sheep were not fed any additional feed rations or supplementary diet substances during the research period. All sheep were drenched with Pyramid (av. liquid 10mL) and Dynamax Controlled Release Capsules 3 times during the research period (January–post annual shearing, April–at time of ram introduction and September pre-lambing).

### Wool sample collection

Wool samples were collected during shearing events that occur as part of the standard operations of merino sheep farming. The wool was removed from the animal by a specialist sheep shearer using an electric Heiniger shearing platform and shearing hand-piece (Shearing Supplies NSW Pty Ltd, Australia).

The animals had previously been shorn in May 2016 at seven to eight months old. The January shearing provided a base line assessment of non-joined merino sheep aged fourteen to fifteen months of age with eight months wool.

The samples were collected while each ewe sheep (n = 46) was being shorn; it was then wrapped inside aluminum foil and placed into a labelled Ziplock bag, and frozen at -10 ^o^C. The sheep’s identification number, site name, shearing type and collection date was then recorded on the bag. Samples were collected from the ewe during pre-joining (fleece shoulder area), gestation (top knot) and post lambing (top knot), as shown in Table 1. All samples were transferred to the laboratory at Western Sydney University (WSU) within four months post collection. The samples were then placed into a -80 ^o^C freezer until analysis. Visual greasy raw wool characteristics including wool length, discoloration or grass vegetable matter content, was not recorded at time of collection.

**Table 1:** Shows the wool sampling dates and morphometric, reproductive characteristics of the merino ewe sheep (n = 46).

Date	Shearing Type	Sheep age	Breeding status	Wool Cortisol (ng/g)	Wool Progesterone (ng/g)
**1/1/2017**	Main shear	14–15 months old	Maiden/Prior to ram being put out	1.33 ± 0.12	0.04 ± 0.005
**16/9/2017**	Top Knot	22–23 months old	Late gestation/ approximately 2 weeks prior to parturition	3.59 ± 0.12	5.53 ± 0.13
**9/12/2017**	Top Knot	24–25 months old	Post lambing/ 2-month-old lambs at foot	3.27 ± 0.14	0.05 ± 0.003

### Sample preparation

Wool samples were processed and analysed at the Stress Laboratory, Hawkesbury, Western Sydney University.

A randomized subsection of the overall sample was selected by the technician pre sample preparation. The sample was laid out on the table and the technician removed 50mg of sub sample fibre.

Wool samples were washed using 90% isopropanol and air dried at room temperature for no longer than 3 days. Weighed sub-samples of wool (50mg) were cut into fine sections (< 5 mm) using a pair of scissors and 90% ethanol was added to each sample in a labelled 1mL Eppendorf tube to enable steroid extraction to occur. Samples remained immersed in 90% ethanol for 3 days for extraction to be completed.

### Enzyme-Immunoassays

The wool cortisol concentration in each sample was determined by colourmetric analysis using polyclonal anticortisol antiserum (R4866 –supplier UC Davies California) diluted in ELISA coating buffer (Carbonate-Bicarbonate Buffer capsule (Sigma C-3041) and 100 mL Milli-Q water, pH 9.6), working dilution 1:15,000. This was followed by reactivity with Horseradish Peroxidase (HRP) conjugated cortisol label (CJM, UC Davies) diluted 1:80,000, and cortisol standards diluted serially (1.56–400 pg/well). Wool progesterone levels were determined using a monoclonal anti-progesterone antiserum (CL425) diluted 1:15,000, horseradish peroxidase conjugated progesterone label (CJM, UC Davies) diluted 1:40,000 and progesterone standards (0.39–100 pg/well).

Nunc Maxi-Sorp plates (96 wells) were coated with 50 μL cortisol antibody solution and incubated for a minimum of 12 hours at 4 ^o^C. Standards, including zeros and nsbs (non-specific binding wells), were prepared serially (2-fold) using 200 μL standard working stock and 200 μL assay buffer (39 mM NaH_2_PO_4_H_2_O, 61 mM NaHPO_4_, 15 mM NaCl).

For all assays, 50 μL of standard and (1:10) diluted 90% ethanol extracted wool samples were added to each well, followed by 50 μL of the cortisol HRP. Each plate was loaded in under 10 minutes. Plates were covered with acetate plate sealer and incubated at room temperature for 2 hours. After incubation, plates were washed 4 times using an automated plate washer (ELx50, BioTek) with phosphate-buffered saline solution (0.05% Tween 20) and then blotted on paper towel to remove any excess wash solution. Substrate buffer was prepared by combining 1 μL 30% H_2_O_2_, 75 μL 1% tetramethylbenzidine (TMB) and 7.425 μL 0.1 M acetate citrate acid buffer, pH 6.0 per plate. The TMB substrate was added to each well that contained a standard sample at 50 μL to generate colour change. The plates were covered with an acetate plate sealer and left to incubate at room temperature for 15 minutes. The reaction was stopped with 50 μL of Stop solution (0.5 M H_2_SO_4_ and Milli-Q water) added to all wells in the Nunc Maxi-Sorp plates. To determine hormone concentration in each sample plates were read at 450nm (reference 630nm) on an ELx800 (BioTek) microplate reader. Wool cortisol and progesterone concentrations were presented as ng/g of dry wool weight.

### Statistical analysis

Statistical analysis and graphs were done using SYSTAT version 13.0. Statistical analysis was done to test the hypothesis that (1) wool cortisol and progesterone levels will be significantly related to the stages of breeding in the merino ewes. Firstly, hormone data checked for homogeneity of variances and were log-transformed prior to analysis. Statistical analysis was done using a repeated measures Analysis of Variance (ANOVA) model using wool sample, date of sampling and sheep number as the factors and log-transformed cortisol and progesterone hormone data as the dependent variables. Level of significance for all statistical analysis was p *<* 0.05. Please refer to S1 Table for raw hormonal data.

## Results

### Wool cortisol and progesterone before, during and after pregnancy in merino ewes

**Fig 1:**
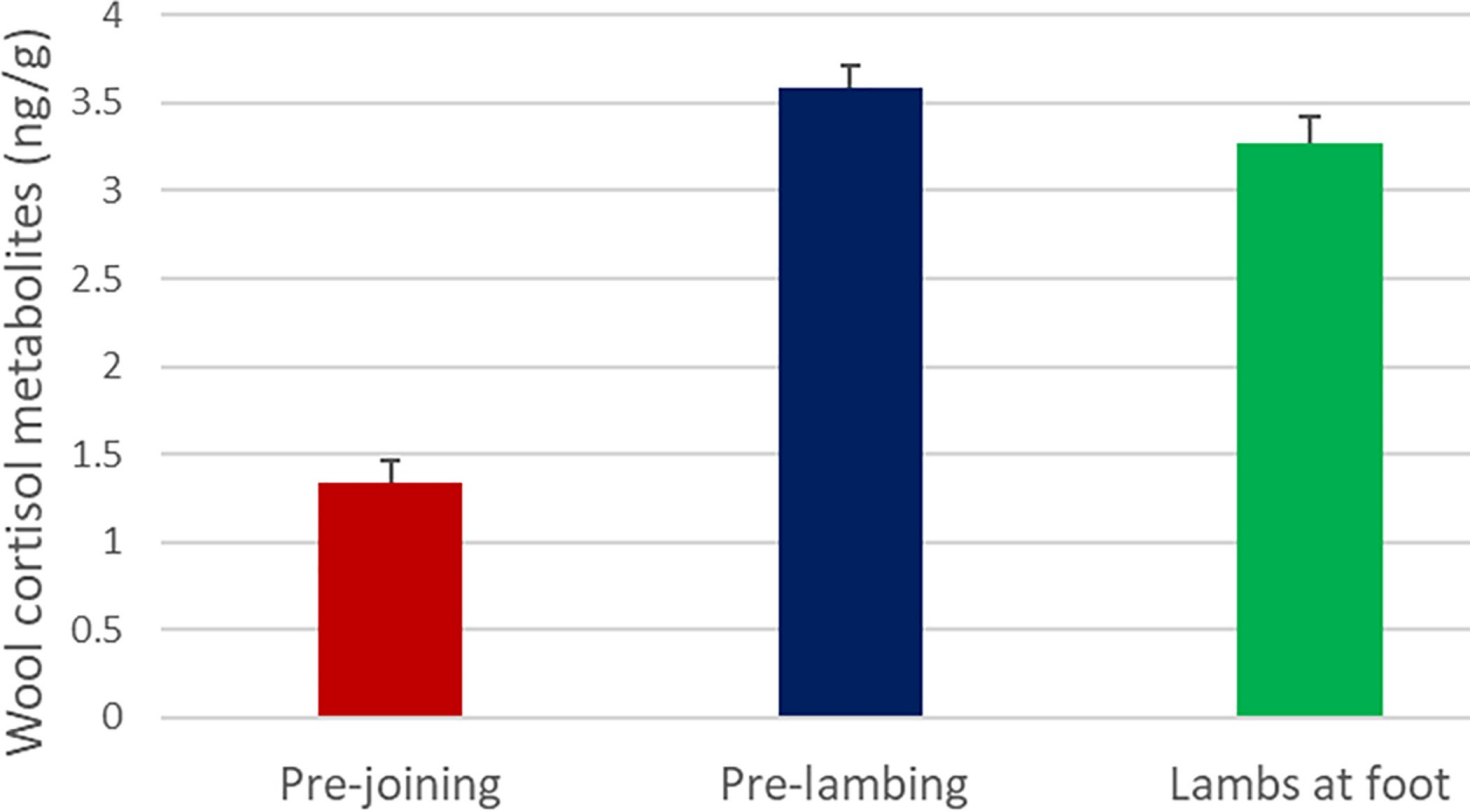
Merino ewe (n = 46) wool cortisol levels before, during and after pregnancy. Wool cortisol levels pre-joining showed significantly lower than levels for pre-lambing and lambs at foot. No significant difference in levels was shown between pre-lambing and lambs at foot.

**Fig 2:**
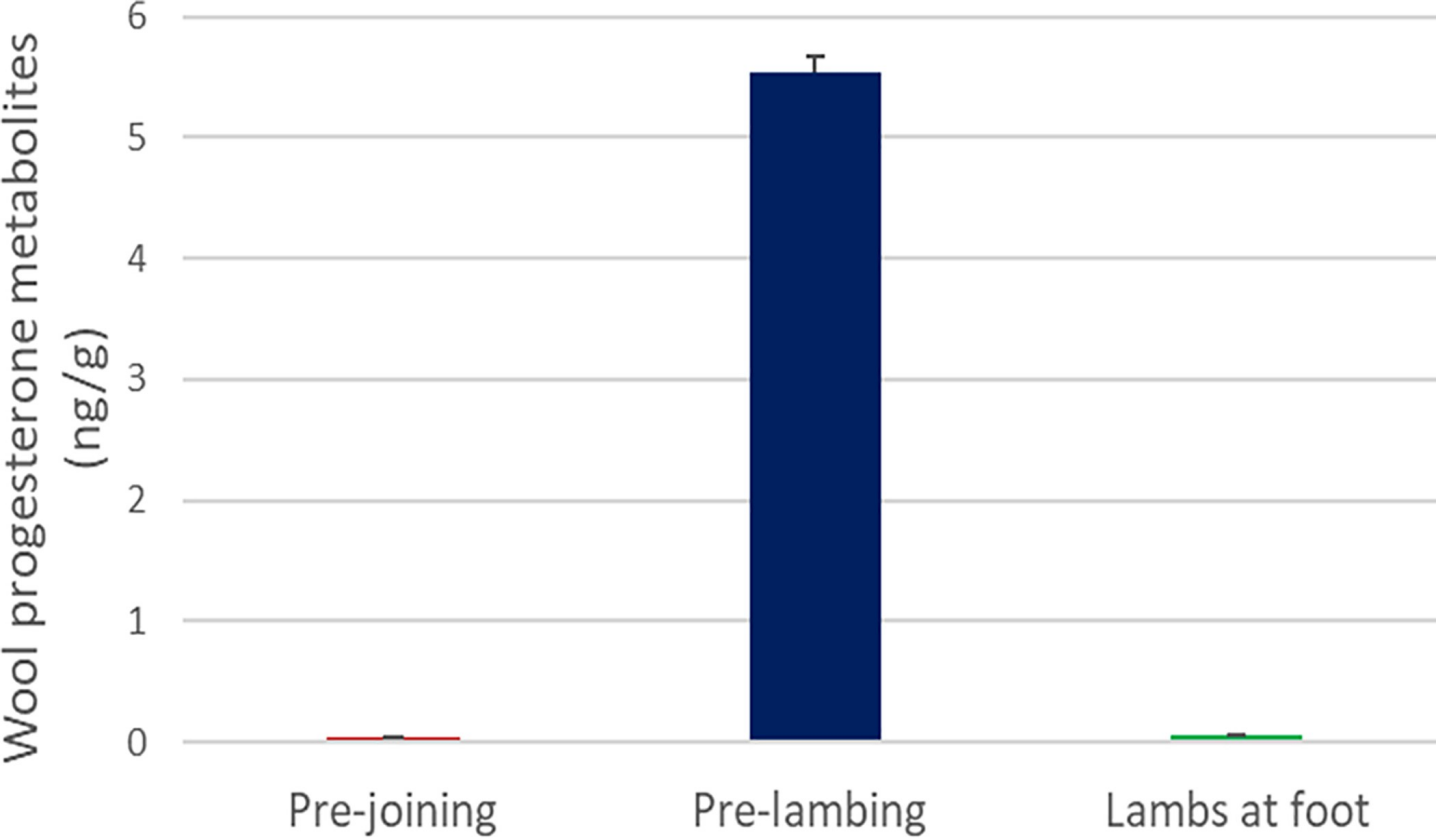
Merino ewe (n = 46) wool progesterone levels before, during and after pregnancy. Wool progesterone levels pre-lambing showed significantly higher than for pre-joining and lambs at foot. No significant difference was shown between pre-joining and post lambing.

Wool concentrations of cortisol and PG of Merino ewes throughout the experiment are depicted in Table 1, Figs 1 and 2.

Wool cortisol levels increased significantly (p = 3.04E-14) during pregnancy (pre-lambing) (Fig 1; Table 1). Levels did not significantly (p = 0.124) decrease post-lambing (lambs at foot). Post lambing (lambs at foot) levels remained significantly (p = 3.82E-10) higher than for pre-joining.

Pregnancy (pre-lambing) significantly (p = 1.83E-33) increased the wool progesterone level in the merino ewes from baseline (pre-joining) levels (Fig 2; Table 1). After parturition (lambs at foot), progesterone levels significantly (p = 5.44E-59) decreased (Fig 2). The pre-conception (pre-joining) and post lambing (lambs at foot) progesterone levels showed a non-significant trend (p = 0.057) (Fig 2).

## Discussion

The present study assessed long term variations of cortisol and PG levels in maiden merino ewes before, during and after pregnancy using wool as a non-invasive hormone assessment tool. Pregnancy in merino ewes elicited significant increases in wool progesterone and cortisol levels. While the PG levels decreased significantly following parturition, cortisol levels did not. To our knowledge, very few studies have used wool to assess long term change in cortisol levels [10, 11]. Furthermore, an assessment of 545 records on google scholar on the 2^nd^ of December 2018 provided no previous published study has used wool to assess long-term progesterone levels in the maiden merino ewes or in maiden ewes from other breeds of sheep. The results of our study are consistent with the predicted outcomes based on the existing literature that have used blood plasma to assess the levels of wool cortisol and progesterone in pregnant ewe sheep.

Regulation of cortisol levels throughout gestation is essential in the production of healthy offspring. Stress-induced elevation of cortisol can negatively impact fertility and fetal health increasing the risk of abortion, underweight neonates and higher levels of cortisol and HPA activity of the offspring at birth and maturity [13–15]. Throughout pregnancy, however, basal plasma cortisol levels must progressively increase to accommodate the nutritional demands of the developing embryo and assist in the development of fetal organs [16, 17]. Maternal plasma cortisol concentrations in sheep can be elevated by three times that of nonpregnant ewes in the later stages of pregnancy [18], with HPA-placental axis activation contributing to these levels [13]. Our results using wool cortisol analysis support the previous research in blood as we found pre-pregnancy cortisol levels increasing 2.70-fold in the later stages of pregnancy in merino ewes. Therefore, our data provides strong support for the use of wool as a non-invasive assessment tool for stress and reproductive hormone measurement in Merino ewes.

Studies conducted by [19–21] showed the concentrations of blood levels of progesterone in sheep before, during and after gestation in sheep. These three studies show a clear trend for increasing circulatory progesterone levels throughout gestation with a sharp decline immediately prior to, and after parturition. These three studies used blood plasma to assess progesterone and it seems that the present study is the first to use wool. The pattern of progesterone levels shown in our wool analysis correlate directly to those shown in the traditional blood sample analysis, giving support to our results and thus the use of wool as a non-invasive method of reproductive hormone assessment.

### Study limitation

While the use of wool has been identified as a reliable indicator of long-term cortisol levels in sheep. It is not possible to stipulate pregnancy as the only variable affecting wool cortisol concentrations. Other environmental factors include access to water, nutrition, climate and predation have also been shown to affect long-term cortisol concentrations [10, 17, 22] and cannot be excluded as potential extrinsic influences within the present study.

## Conclusion

Non-invasive assessment techniques of stress and reproductive hormones used in this study have many potential applications in future livestock research. The greatest potential, however, lies in the development of a commercially viable adaptation of this assessment technique that can be applied to industry management. Regular testing would be key to identify baseline levels for individual ewes and allow early detection of physiological stress that the sheep maybe experiencing. Identification of individuals with elevated concentrations combined with poor health and/or poor fertility can also assist farmers in selecting the best stock to retain and breed from, hence supporting animal welfare requirements.

## Supporting information

S1 Table Raw data for reproductive and stress hormones. (XLSX)
